# Stroke Preventability in Large Vessel Occlusion Treated With Mechanical Thrombectomy

**DOI:** 10.3389/fneur.2021.608084

**Published:** 2021-03-08

**Authors:** Shuichi Suzuki, Lara Wadi, Lisa Moores, Ichiro Yuki, Jeein Kim, Jordan Xu, Annlia Paganini-Hill, Mark Fisher

**Affiliations:** ^1^Department of Neurological Surgery, University of California, Irvine, Irvine, CA, United States; ^2^Department of Neurology, University of California, Irvine, Irvine, CA, United States; ^3^UC Irvine Medical Center, Orange, CA, United States

**Keywords:** stroke, prevention, treatment, thrombectomy, outcome

## Abstract

**Objective:** The preventability of strokes treated by mechanical thrombectomy is unknown. The purpose of this study was to analyze stroke preventability for patients treated with mechanical thrombectomy for large vessel occlusion.

**Methods:** We conducted retrospective analyses of 300 patients (mean ± SE age 69 ± 0.9 years, range 18–97 years; 53% male) treated with mechanical thrombectomy for large vessel occlusion from January 2008 to March 2019. We collected data including demographics, NIH Stroke Scale (NIHSS) at onset, and (beginning in 2015) classified 90-day outcome by modified Rankin Scale (mRS). Patients were evaluated using a Stroke Preventability Score (SPS, 0 to 10 points) based on how well patients had been treated given their hypertension, hyperlipidemia, atrial fibrillation, and prior stroke history. We examined the relationship of SPS with NIHSS at stroke onset and with mRS outcome at 90 days.

**Results:** SPS was calculated for 272 of the 300 patients, with mean ± SE of 2.1 ± 0.1 (range 0–8); 89 (33%) had no preventability (score 0), 120 (44%) had low preventability (score 1–3), and 63 (23%) had high preventability (score 4 or higher). SPS was significantly correlated with age (*r* = 0.32, *p* < 0.0001), while NIHSS (*n* = 267) was significantly higher (*p* = 0.03) for patients with high stroke preventability vs. low/no preventability [18.8 ± 0.92 (*n* = 62) vs. 16.5 ± 0.51 (*n* = 205)]. Among 118 patients with mRS, outcome was significantly worse (*p* = 0.04) in patients with high stroke preventability vs. low/no preventability [4.7 ± 0.29 (*n* = 28) vs. 3.8 ± 0.21 (*n* = 90)]. The vast majority of patients with high stroke preventability had inadequately treated atrial fibrillation (85%, 53/62).

**Conclusions:** Nearly one quarter of stroke patients undergoing mechanical thrombectomy had highly preventable strokes. While stroke preventability showed some relationship to stroke severity at onset and outcome after treatment, preventability had the strongest association with age. These findings emphasize the need for improved stroke prevention in the elderly.

## Introduction

Mechanical thrombectomy (MT) for acute ischemic stroke due to large vessel occlusion (LVO) is the standard of care in appropriately selected patients (1). LVO comprises nearly half of acute ischemic stroke, contributes disproportionately to poor functional outcome, and successful endovascular MT significantly improves that outcome (2, 3). As a consequence, stroke systems of care to disseminate MT to achieve reperfusion in ischemic brain tissue have been developed (1, 2).

Attempts to improve outcome after MT for LVO have focused on improved workflow to reduce or eliminate any delay in initiating treatment. In other words, the emphasis has been on establishing the *treatability* of acute LVO and beginning MT as soon as possible, with the expectation that improving the logistics of MT is the best strategy for improving stroke outcome. There is, in fact, no serious reason to question this strategy. Nevertheless, other issues may importantly contribute to stroke outcome.

Stroke treatment and stroke prevention may be considered as distinct and perhaps unrelated entities. We previously demonstrated an unexpected relationship between stroke treatability and stroke *preventability*, with acute stroke treatment directed disproportionately toward patients with a high degree of preventability (4). In the current study, we analyzed the prevalence and consequences of stroke preventability in a cohort of patients undergoing MT for LVO. We hypothesized that the preventability of ischemic stroke was related to stroke severity and stroke outcome.

## Methods

### Patients

We reviewed medical records of patients who underwent MT at our facility for the treatment of acute ischemic stroke between January 1, 2008 and March 10, 2019. Data collection was conducted from January 28, 2019 to July 17, 2019. There were no exclusion criteria. We collected information on patient demographics, medical history, medication history, laboratory values, vital signs, and NIH Stroke Scale (NIHSS) on presentation of stroke. For the patients seen in 2015 or later, we used the modified Rankin Scale (mRS) to classify their 90-day outcome. The study was approved by the University of California Irvine Institutional Review Board, which waived the need for informed consent.

### Stroke Preventability Score

Patients were evaluated using a Stroke Preventability Score (SPS) (4). The SPS addresses the extent of stroke preventability using four vascular risk factors (blood pressure, cholesterol level, atrial fibrillation, and prior stroke, transient ischemic attack or myocardial infarction) and their treatment and is scored 0 to 10 (Table 1). SPS was categorized as no preventability (score of 0), low preventability (1–3), and high preventability (4 or higher). Blood pressure was considered poorly treated if at the time of stroke presentation systolic blood pressure was 200 mmHg or higher (2 points), suboptimally treated if between 180 to 199 mmHg (1 point), or adequately treated if <180 mmHg (0 points). Cholesterol was considered poorly treated if at the time of stroke presentation total cholesterol was 200 mg/dL or higher or low-density lipoprotein cholesterol was 150 mg/dL or higher (2 points); suboptimally treated if total cholesterol was between 180 to 199 mg/dL or low-density lipoprotein cholesterol between 100 to 149 mg/dL (1 point); and adequately treated if total cholesterol was <180 mg/dL and low-density lipoprotein cholesterol <100 mg/dL (0 points). Patients with a history of or presenting with atrial fibrillation and not receiving anticoagulant medication (owing to interruption in treatment, noncompliance, or medication not prescribed) were considered untreated (4 points). Patients with a history of atrial fibrillation who were taking an anticoagulant and had an international normalized ratio of <2 were considered suboptimally treated (2 points). Patients with a history of atrial fibrillation and an international normalized ratio of 2 or higher, taking a new-generation anticoagulant (not warfarin), not presenting with atrial fibrillation on admission, or having no history of atrial fibrillation were considered optimally treated (0 points). Patients with a prior stroke, transient ischemic attack, or myocardial infarction and not taking platelet medications were considered untreated (2 points); patients with a similar history and additional history of atrial fibrillation without therapeutic anticoagulation were also given 2 points; patients with a history of stroke, transient ischemic attack, or myocardial infarction and taking platelet medications or receiving therapeutic anticoagulation treatment were considered optimally treated (0 points). Patients with no history of stroke, transient ischemic attack, or myocardial infarction were also given 0 points.

**Table 1:** Classification of stroke preventability scores (SPS) for each vascular comorbidity.

**Comorbidity**	**Score**	**Classification**
Hypertension	2	SBP: ≥200 mmHg
	1	SBP: 180–199 mmHg
	0	SBP: <180 mmHg
Dyslipidemia	2	Total cholesterol: ≥200 mg/dL or LDL: ≥150 mg/dL
	1	Total cholesterol: 180–199 mg/dL or LDL: 100–149 mg/dL
	0	Total cholesterol: <180 mg/dL and LDL: <100 mg/dL
Atrial fibrillation	4	History of or presenting with atrial fibrillation not on anticoagulation medication
	2	History of atrial fibrillation with an INR: <2 on anticoagulation medication
	0	History of atrial fibrillation with an INR: ≥2, on new-generation anticoagulation medication, or no history of atrial fibrillation
Vascular history	2	History of stroke, TIA, or MI not on antiplatelet medication, or history of stroke, TIA, or MI with history of atrial fibrillation not on anticoagulation medication
	0	History of stroke, TIA, or MI on antiplatelet or anticoagulation medication, or no history of stroke, TIA, or MI

### Statistical Analysis

Means and standard errors (SE) of continuous variables and proportions of qualitative variables are presented. Differences between stroke preventability groups (low, SPS <4 vs. high, SPS 4+) were tested using Krushal-Wallis test for continuous variables (age, NIHSS, and mRS) and Fisher's exact test for categorical variables of stroke severity (mild-moderate stroke, NIHSS <16 vs. severe stroke, NIHSS 16+) and stroke outcome (good outcome, mRS <3 vs. poor outcome, mRS3+). Spearman rank correlation coefficients were calculated for SPS with age, NIHSS, and mRS.

## Results

Three-hundred stroke patients were treated with MT for LVO between January 1, 2008 and March 10, 2019. The characteristics of the 300 stroke patients seen between 2008 and 2019 are shown in Table 2. Patients ranged in age from 18 to 97 with mean of 69; 53% were male, and M1 segment of the middle cerebral artery was the most common site of occlusion (45%). MT was performed using various devices, including the first-generation Mechanical Embolus Removal in Cerebral Ischemia (MERCI) Retriever to the most advanced stent retriever devices and A Direct Aspiration First Pass Technique (ADAPT), or a combination of those. Mean onset-to-puncture, door-to-puncture, onset-to-recanalization, and puncture-to-recanalization times are shown in Table 3.

**Table 2:** Characteristics of 300 stroke patients.

**Age in years**	
Mean ± SE	69 ± 0.9
Range	18 to 97
**Male sex**	160 (53%)
**Race**	
White	182 (61%)
Black	6 (2%)
Asian	75 (25%)
Not specified or other	37 (12%)
**Arterial occlusion site**	
Basilar	41 (14%)
Middle Cerebral Artery	
M1	136 (45%)
M2	34 (11%)
M3	4 (1%)
Internal Cerebral Artery	51 (17%)
Tandem	23 (8%)
Post-Tandem	1 (0.3%)
Posterior Cerebral Artery	6 (2%)
Vertebral Artery	1 (0.3%)
Anterior Cerebral Artery	2 (0.7%)
Unknown	1 (0.3%)
**NIHSS**	(*n* = 284)
Mean ± SE	17 ± 0.4
Range	0 to 37
**Stroke preventability score**	(*n* = 272)
0	89 (33%)
1	40 (15%)
2	66 (24%)
3	14 (5%)
4	19 (7%)
5	6 (2%)
6	22 (8%)
7	14 (5%)
8	2 (1%)
Mean ± SE	2.1 ± 0.1
**mRS at 90 days**	(*n* = 118)
0	3 (3%)
1	14 (12%)
2	15 (13%)
3	17 (14%)
4	9 (8%)
5	18 (15%)
6	42 (36%)
Mean ± SE	4.0 ± 0.2


**Table 3:** Time windows for mechanical thrombectomy.

	**Number of patients with complete data**	**Mean ± SE (minutes)**
Onset-to-puncture	194	300 ± 12
Door-to-puncture	200	142 ± 7
Onset-to-recanalization	149	392 ± 15
Puncture-to-recanalization	196	99 ± 5

**Figure 1:**
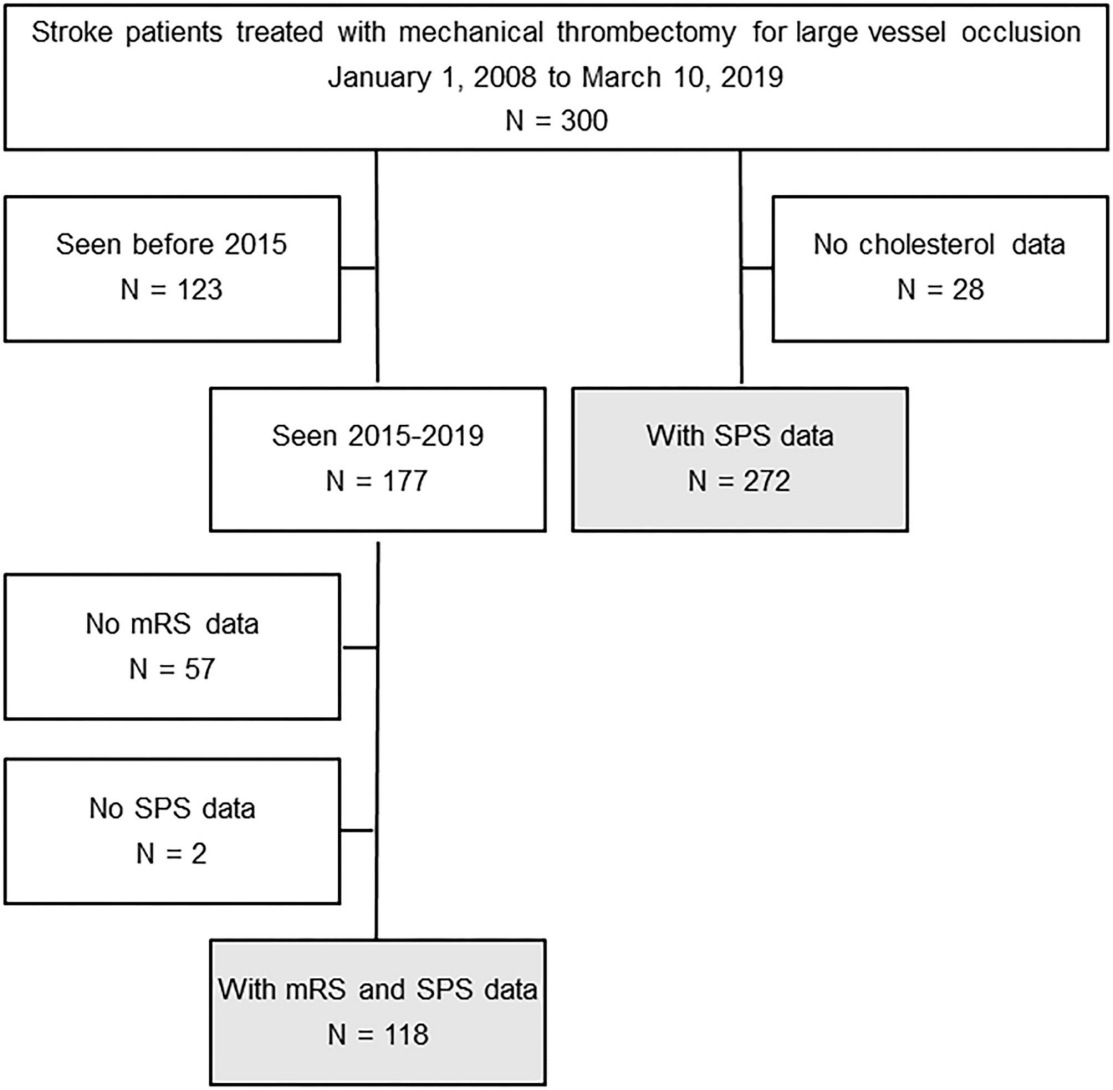
Consort diagram.

Among these patients, 272 had SPS data, and 118 had both mRS and SPS data (Figure 1). Among the 284 (of the total 300) with NIHSS score, the mean was 17 (range 0 to 37). Among the 272 with SPS (missing in 28 due to lack of cholesterol levels), the mean was 2.1; 89 (33%) had no preventability (score 0), 120 (44%) had low preventability (score 1-3), and 63 (23%) had high preventability (score 4+). Among the 300 patients in this study, 161 (54%) received intravenous tissue plasminogen activator (IV tPA). Among the 272 patients with SPS data, mean SPS (2.1 vs. 2.1) and the proportion with high preventability scores (scores 4+) (25 vs. 21%) did not differ between those given tPA and those not.

Of the 63 patients with high preventability, 54 (86%) had an atrial fibrillation prevention score of 4, 33 (52%) a vascular history preventability score of 2, 14 (22%) a hypertension preventability score of 2, 6 (10%) a dyslipidemia preventability score of 2. The patients in the high preventability group had significantly (*p* = 0.03) more severe deficit on presentation [NIHSS 16.5 ± 0.51 (*n* = 205) vs. 18.8 ± 0.92 (*n* = 62)]; this association remained significant (*p* = 0.01) when the blood pressure control SPS variate was excluded from the analysis. Comparison using dichotomized classification of NIHSS again showed significant (*p* = 0.03) associations between SPS and NIHSS: severe strokes were seen in 74% (46/62) of the high preventability group compared with 58% (119/205) in the low preventability group. SPS was significantly associated with age (*r* = 0.32, *p* < 0.0001).

Of the 177 stroke patients seen in 2015 or later, 120 had mRS score at 90 days, but two did not have SPS. The characteristics of the remaining 118 patients are similar to all patients. Age ranged from 25 to 97 with mean of 70; 54% were male. The mean NIHSS score was 17 (range 1 to 36). The mean SPS was 2.1; 39 (33%) had no preventability (score 0), 51 (43%) had low preventability (score 1-3), and 28 (24%) had high preventability (score 4+). The mean mRS was 4.0. The patients in the high preventability group had significantly greater disability at 90 days (mRS 3.8 ± 0.21 vs. 4.7 ± 0.29, *p* = 0.04); this trend remained with removal of blood pressure control SPS variate, but was no longer statistically significant (*p* = 0.11). Comparison using dichotomized classification of mRS again showed a significant (*p* = 0.03) association between SPS and mRs: poor outcome was present in 89% (25/28) of patients in the high preventability group compared with 68% (61/90) of those in the low preventability group.

Among the 118 patients with complete data on SPS, NIHSS and mRS, SPS was significantly correlated with age (r=0.33, *p* = 0.0003), mRS (*r* = 0.20, *p* = 0.03) and NIHSS (*r* = 0.18, *p* = 0.047). Only the partial correlation coefficients of SPS with age (*r* = 0.26, *p* = 0.005), adjusting for the other two variables, remained statistically significant. The two SPS groups (no or low vs. high) differed significantly in age (*p* = 0.001), NIHSS (*p* = 0.02), and mRS (*p* = 0.04) (Table 4). The patients in the high preventability group were on average older, had more severe deficit on presentation (NIHSS) and had greater disability at 90 days (mRS). Of the 28 patients with high preventability, all but 3 were aged 70+; of those aged 70+ with high preventability, 88% had atrial fibrillation scores of 4.

**Table 4:** Preventability score category by age, NIHSS, and mRS^†^.

	**Preventability Score**		
	**0-3**	**4+**	*P*-value^*^
Number	90	28	
	**Mean** **±** **SE**	**Mean** **±** **SE**	
Age	68 ± 1.7	79 ± 1.9	0.001
NIHSS	16.0 ± 0.8	20.3 ± 1.4	0.02
mRS at 90 days	3.8 ± 0.3	4.7 ± 0.3	0.04

† *for 118 patients with complete data on SPS, NIHSS and mRS*.

* *statistical difference between group of no/low preventability score (0–3) and high preventability score (4+) using Krushal-Wallis test*.

## Discussion

This retrospective study analyzed stroke preventability in a group of acute ischemic LVO stroke patients who underwent MT over a 10-year period. Consistent with our prior study (4), our findings indicate that a large proportion of these patients had strokes that were preventable to some extent, and that nearly one quarter had highly preventable strokes. Stroke preventability was most strongly associated with increasing age, and on univariate analysis predicted both severity of stroke at onset and outcome after MT.

While the vast majority of our patients had strokes that were preventable to some extent, the role of atrial fibrillation is particularly notable. Stroke prevention in atrial fibrillation has historically been problematic, with some studies showing untreated or poorly treated atrial fibrillation in ~40% of cases and with little relationship between anticoagulation usage and stroke risk (5–13). Our findings emphasize the consequences of inadequate treatment of atrial fibrillation, which importantly contributes to the extent of stroke preventability in patients undergoing MT.

Our data emphasize the overarching importance of age in stroke prevention. Stroke preventability was most significantly associated with increasing age, suggesting that this population deserves enhanced attention for stroke prevention efforts. Aged patients tend to do less well after MT (14), and thus the issue of improving stroke prevention for this group takes on added urgency. This is in line with existing literature that suggests that not only does the rate of ischemic strokes increase with age, but that older patients also tend to have more disability after thrombectomy (15). This was also reflected in the HERMES study, where lower 90-day mRS scores were reported in younger patients, suggesting that age could be a negative predictor of outcome after MT (16).

It may be tempting to view stroke treatment and stroke prevention as separate entities. This is an understandable response to the exigencies of acute treatment efforts, in which “time is brain” and all attention is given to the scope of treatability of the acute stroke patient. Nevertheless, our data argue that to some extent, stroke prevention and stroke treatment are inter-related. Our univariate analysis found that not only was the neurological deficit on presentation reflective of the preventability of the stroke, but also that the outcome after MT was to some extent predictable based on the preventability of the very stroke that was treated.

Our study has several limitations. The SPS was not intended to incorporate all stroke risk factors, as this study was designed primarily as a conceptual exploration of the relationship between stroke prevention and stroke treatment. Risk factors such as smoking and diabetes were not included. Note however that while control of diabetes is indeed modifiable, diabetes control has generally not been clearly tied to stroke risk (17), making inclusion of diabetes into the SPS problematic. Smoking was not included in the SPS in part because discontinued smoking remains a vascular risk for 10–15 years (18), making smoking discontinuation less relevant for stroke preventability in this context. Finally, blood pressure measurement at the time of acute stroke may not necessarily reflect blood pressure control prior to the event. Nevertheless, we observed similar trends when the blood pressure control variate was not included in the analysis.

In conclusion, a large proportion of patients presenting with acute ischemic stroke secondary to LVO and treated with MT had strokes that were preventable to some extent. High stroke preventability was encountered in nearly one quarter of patients and was substantially driven by inadequately treated atrial fibrillation. Stroke preventability was greatest in the elderly, and preventability tended to predict both the severity of the presenting stroke and their outcome following MT. These findings lend some support to initial observations linking the preventability and treatability of acute strokes, and raise the perhaps paradoxical notion that efforts to improve outcome after MT may benefit by incorporating improved stroke prevention programs. The elderly appear to be potentially the greatest beneficiaries of improved stroke prevention, and specific strategies to achieve this warrant further attention.

## Data Availability Statement

The raw data supporting the conclusions of this article will be made available by the authors, without undue reservation.

## Ethics Statement

The studies involving human participants were reviewed and approved by University of California, Irvine Institutional Review Board. Written informed consent for participation was not required for this study in accordance with the national legislation and the institutional requirements. Requirement for obtaining informed consent waived for this study by the University of California Institutional Review Board.

## Author Contributions

SS contributed to conception and design of the work, interpretation of data, and drafting the manuscript. LW contributed to acquisition of data, interpretation of data, and drafting the manuscript. LM contributed to conception and design of the work, acquisition and interpretation of data, and revising the manuscript. IY contributed to acquisition of data and revising the manuscript. JK contributed to acquisition of data and revising the manuscript. JX contributed to acquisition of data and revising the manuscript. AP-H contributed to conception and design of the work, acquisition, analysis and interpretation of the data, and drafting the manuscript. MF contributed to conception and design of the work, interpretation of data, and drafting the manuscript.

## Conflict of Interest

The authors declare that the research was conducted in the absence of any commercial or financial relationships that could be construed as a potential conflict of interest.
